# Management of neonatal pyonephrosis in a low-resource setting: a case report

**DOI:** 10.1097/MS9.0000000000005057

**Published:** 2026-06-03

**Authors:** Nabin Adhikari, Diwakar Koirala, Ramesh Sapkota, Bivek Mishra, Niraj Paudel, Deepak Mishra

**Affiliations:** Department of Pediatrics, B.P. Koirala Institute of Health Sciences, Dharan, Nepal

**Keywords:** Acinetobacter baumannii, infant, low-resource setting, pyonephrosis

## Abstract

**Introduction and importance:**

Pyonephrosis is a urological emergency characterized by the presence of purulent material within an obstructed renal collecting system, often leading to rapid parenchymal destruction and systemic sepsis. While it is well documented in adults and older children, its occurrence in neonates is exceedingly rare. Early diagnosis and timely drainage are essential to prevent long-term renal impairment or mortality, particularly in low-resource settings where access to advanced imaging and pediatric urology is limited.

**Case presentation:**

We report the case of a 48-day-old male infant who presented to the pediatric emergency department with a 5-day history of undocumented fever, persistent vomiting, shrill crying, and progressive abdominal distension. Physical examination revealed pallor and mild abdominal distension. Laboratory tests showed leukocytosis with neutropenia and pyuria. Ultrasonography revealed significant left-sided hydronephrosis with echogenic debris in the pelvicalyceal system, suggestive of pyonephrosis. An emergency ultrasound-guided percutaneous nephrostomy was performed, which drained approximately 15 mL of thick purulent fluid. Culture of the nephrostomy aspirate revealed the *Acinetobacter baumannii* complex, resistant to piperacillin and sensitive to cefotaxime and amikacin.

**Clinical discussion:**

The infant was initiated on empirical antibiotics, which were later tailored to intravenous ampicillin and amikacin based on sensitivity results. Within 2 days, the patient showed marked clinical improvement, with normalization of vital signs, improved oral intake, and resolution of fever. Repeat urine cultures were sterile, and the nephrostomy tube was removed after imaging confirmed resolution. The infant was discharged in stable condition with preserved renal function.

**Conclusion:**

This case underscores the importance of maintaining a high index of suspicion for pyonephrosis in neonates presenting with non-specific symptoms such as fever and irritability. In the absence of advanced imaging, bedside ultrasonography proved invaluable for timely diagnosis. Despite the presence of a multidrug-resistant organism, targeted antibiotic therapy and prompt percutaneous drainage led to full recovery. This report highlights that, even in resource-limited settings, favorable outcomes can be achieved through early recognition, essential diagnostics, and minimally invasive interventions.

## Introduction

Pyonephrosis is a suppurative infection of the hydronephrotic kidney, characterized by the accumulation of purulent material in the renal pelvis, leading to parenchymal destruction and loss of renal function. It represents a urological emergency that, if untreated, can lead to rapid deterioration from local infection to systemic sepsis and multiorgan failure^[^1,2^]^. While the condition is documented in adults and older children, its occurrence in neonates is exceedingly rare, with few reported cases in the literature^[^1,3^]^.

HIGHLIGHTS

- This report presents a rare case of acute pyonephritis in a 48-day-old infant.
- The case was managed in low-resource setting without sophisticated tools.
- A high degree of suspicion, rapid initiation of appropriate antibiotics, and drainage of pus are crucial in the management of pyonephrosis.
- In the management of pyonephrosis, the selection of appropriate antibiotics is essential.
- Even though emergency nephrectomy was the standard procedure for pyonephrosis, timely drainage of pus with a percutaneous nephrostomy tube can prevent nephrectomy. Nephrostomy also serves the dual purpose of uncovering the obstructed kidney’s anatomy.
- Ultrasonography has high specificity for the diagnosis of pyonephrosis, although sensitivity seems relatively low.
- Prompt diagnosis, multidisciplinary management, and awareness of local microbial resistance are important for successful treatment.

In neonates, clinical manifestations are often non-specific, such as fever, vomiting, and irritability, making early diagnosis difficult. Moreover, signs of systemic inflammatory response syndrome, including leukocytosis, hypothermia or fever, tachypnea, and altered perfusion, may be masked or delayed in infants^[^2^]^. This contributes to the high risk of delayed intervention and potential for adverse outcomes, including irreversible renal damage or death.

Diagnostic imaging plays a central role in the evaluation of suspected pyonephrosis. Ultrasonography (US) is often the first-line imaging modality due to its availability and non-invasive nature. However, it has limited sensitivity in detecting early renal parenchymal involvement, especially in neonates^[^4,5^]^. Computed tomography (CT) and magnetic resonance urography (MRU) offer greater diagnostic accuracy but may not be feasible in low-resource settings due to availability and the need for sedation in infants^[^4^]^.

The cornerstone of management includes early initiation of empiric antibiotics followed by targeted antimicrobial therapy guided by culture and sensitivity reports. In cases with significant obstruction or sepsis, drainage of pus via nephrostomy or surgical intervention becomes essential. Historically, emergency nephrectomy was often performed, but modern management emphasizes nephron-sparing strategies like percutaneous nephrostomy^[^6^]^.

In resource-constrained environments, managing neonatal pyonephrosis presents unique challenges. Limited access to advanced imaging, delayed laboratory results, and restricted antibiotic choices increase the complexity of care. Moreover, emerging resistance patterns among causative pathogens, such as *Acinetobacter baumannii*, highlight the importance of local surveillance to guide empiric therapy^[^7^]^.

This report presents a rare case of *A. baumannii*–associated pyonephrosis in a 48-day-old infant managed successfully with US-guided percutaneous nephrostomy and targeted antibiotics. It illustrates how timely recognition, multidisciplinary coordination, and reliance on essential tools can lead to favorable outcomes, even in low-resource settings.

The manuscript has been reported in accordance with the TITAN checklist^[^8^]^.

## Case presentation

A 48-day-old male infant, born full-term via normal vaginal delivery with a birth weight of 3.1 kg, was brought to the pediatric emergency department with a 5-day history of undocumented fever, excessive crying, non-bilious vomiting, and abdominal distension. The mother reported that the child had become increasingly irritable, with reduced feeding and fewer wet diapers over the preceding 2 days. On Day 3 of illness, the infant had five episodes of watery stools. No prior antibiotic exposure or hospitalization was reported. The family resides in a rural region with limited access to sanitation and healthcare.

There was no history of hematuria, foul-smelling urine, or visible abdominal mass. The infant was exclusively breastfed, and there was no known history of maternal infections during pregnancy.

On examination at admission, the findings were as follows:

- Weight: 3.7 kg
- Temperature: 38.3°C
- Heart rate: 160 bpm
- Respiratory rate: 52 breaths/min
- Blood pressure: 72/40 mmHg
- SpO_2_: 96% on room air

The child appeared pale, irritable, and mildly dehydrated. Abdominal examination revealed mild distension with no guarding, rigidity, or palpable mass.

Initial laboratory investigations showed the following:

- Total leukocyte count: 11 380/mm^3^
- Neutrophils: 18%
- Lymphocytes: 61%
- Hemoglobin: 10.1 g/dL
- PCV: 29%
- Serum creatinine: 0.5 mg/dL
- Blood urea: 24 mg/dL
- Serum potassium: 5.8 mmol/L
- CRP: 10.2 mg/L
- Urine routine and microscopy examination: pyuria with 20–25 WBCs/hpf

**Figure 1.**
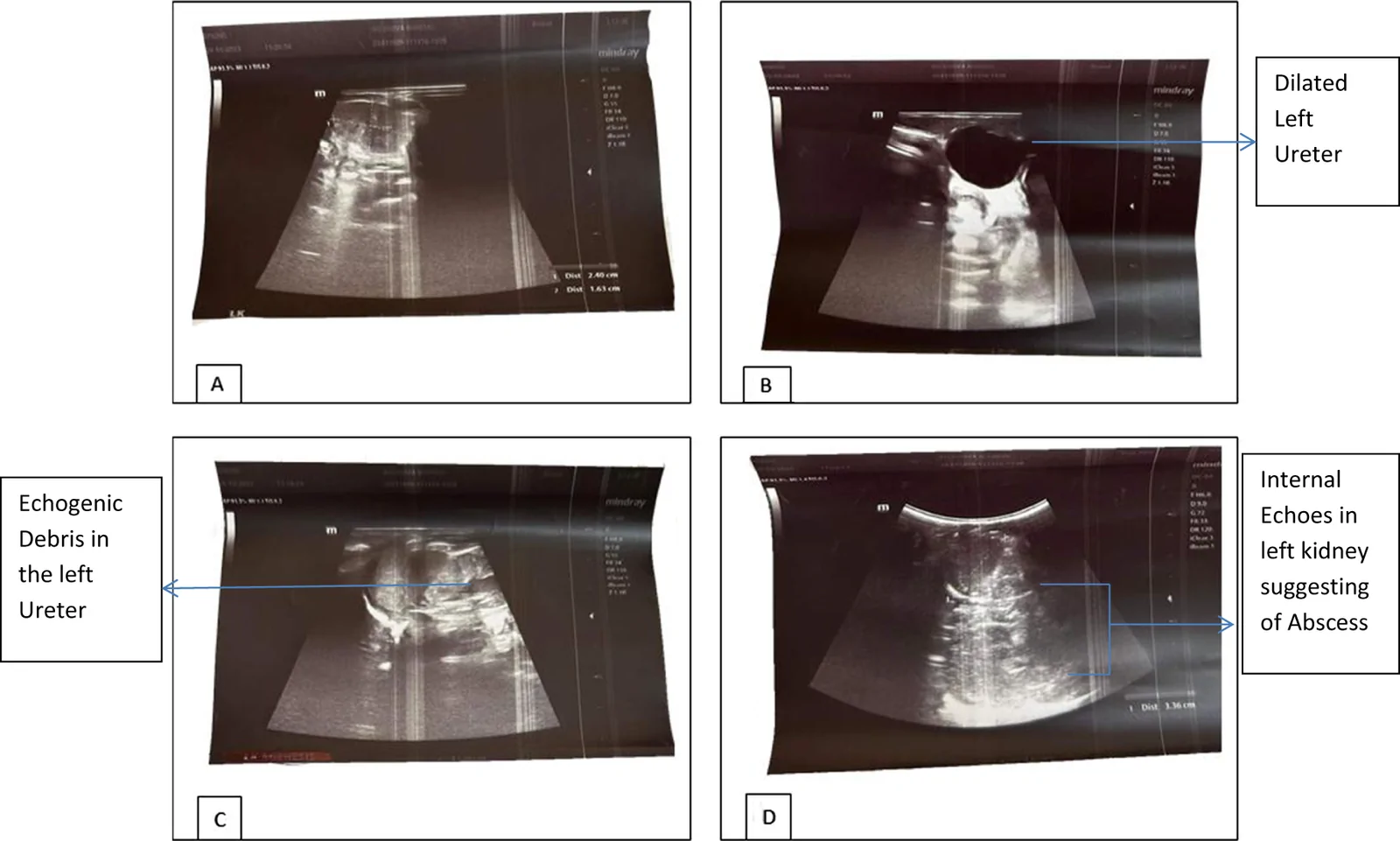
Ultrasonography of the kidney and pelvis showing internal echoes and echogenic debris (arrows) in the left kidney with a dilated ureter (asterisk): pyonephrosis.

Ultrasound of the abdomen and pelvis revealed the following, as shown in Figure 1:

1. Gross left-sided hydronephrosis with significant pelvicalyceal dilation
2. Echogenic debris within the renal pelvis
3. Left ureteral dilation consistent with pyonephrosis

Clinical timeline

Date Event

Day 0 Symptom onset: fever, vomiting, and irritability

Day 5 Hospital presentation and ultrasound diagnosis

Day 5 Emergency percutaneous nephrostomy performed

Day 6 Culture results obtained; antibiotics adjusted

Day 7 Clinical improvement: fever subsided, feeding better

Day 12 Nephrostomy tube removed

Day 15 Patient discharged in stable condition

Day 29 Follow-up ultrasound confirmed full resolution

### Management

An emergency percutaneous nephrostomy was performed under ultrasound guidance, draining approximately 15 mL of thick purulent material. The aspirated fluid was sent for culture and sensitivity testing.

### Microbiology results

The culture of nephrostomy fluid grew the *A. baumannii* complex, resistant to piperacillin but sensitive to cefotaxime and amikacin. Blood cultures were sterile.

Empirical antibiotics were initiated and later switched to IV ampicillin (100 mg TID) and amikacin (30 mg OD) as per sensitivity. By Day 2 of treatment, the infant’s fever subsided, feeding improved, and urine output normalized.

Daily monitoring included fluid balance, vitals, weight, and signs of nephrostomy tube complications. A repeat urine culture on Day 5 was sterile. The nephrostomy tube was removed on Day 7 post-procedure after radiological resolution was confirmed.

### Discharge and follow-up

The infant was discharged on Day 10 of hospitalization in a stable condition. Follow-up US at 2 weeks post-discharge showed normal renal cortical thickness and resolution of hydronephrosis. Growth and development milestones were age-appropriate.

## Discussion

### Pathophysiology

Pyonephrosis refers to a suppurative infection of the renal collecting system, occurring in the setting of urinary tract obstruction. The stagnation of infected urine leads to accumulation of pus, increased pressure within the kidney, and destruction of renal parenchyma. In neonates, risk factors include immature immune responses, congenital urinary tract anomalies, and potential ascending infections. Although urinary tract infections (UTIs) are relatively more common in this age group, progression to pyonephrosis is extremely rare and carries a high risk of sepsis and permanent renal injury if not promptly treated.

### Diagnostic considerations

The diagnosis of pyonephrosis in neonates is particularly challenging due to non-specific clinical signs such as fever, vomiting, irritability, and abdominal distension. These symptoms often overlap with other neonatal conditions, including gastroenteritis, neonatal sepsis, and colic. Physical signs like abdominal mass or flank tenderness may be subtle or absent. In this case, US played a pivotal role by identifying pelvicalyceal dilation and internal echogenic debris, hallmarks of pyonephrosis. Although CT and MRU offer superior anatomical detail, their unavailability in low-resource settings makes point-of-care US a critical tool for timely diagnosis. CT serves as the preferred imaging modality for evaluating most UTI cases due to its rapid acquisition and ability to deliver comprehensive anatomical and physiological insights. It effectively delineates both renal and extra-renal pathologies and enables multiphase assessment following intravenous contrast administration. Additional benefits of CT include its capacity for multiplanar reconstruction, curved planar reformatted imaging, maximum intensity projection, and three-dimensional reconstruction, enhancing diagnostic precision^[^9^]^.

### Differential diagnosis

The clinical presentation of a febrile neonate with vomiting, irritability, and abdominal distension necessitates a broad differential diagnosis due to the non-specific nature of symptoms in early infancy. The following conditions were considered prior to confirming the diagnosis of pyonephrosis:

**Hydronephrosis (non-infective**): It is a non-infective dilation of the renal pelvis and calyces, which typically lacks echogenic debris on ultrasound and may be asymptomatic unless severe. The presence of systemic signs like fever, pyuria, and purulent aspirate excluded simple hydronephrosis in our case.

**Renal or perinephric abscess:** These are localized collections of pus in or around the kidney, which can present with systemic features similar to pyonephrosis. However, imaging usually shows distinct hypoechoic or complex lesions, in contrast to the uniform pelvicalyceal dilation seen in pyonephrosis.

**Neonatal sepsis of unknown source:** Generalized sepsis, particularly from gram-negative organisms, is a key differential. In our patient, the imaging and urine findings localized the infection to the urinary tract, favoring pyonephrosis.

**Gastrointestinal (GI) causes (e.g., Hirschsprung’s disease, malrotation):** These may present with vomiting and abdominal distension. The absence of bilious vomiting, abnormal bowel sounds, and obstructive GI findings on imaging helped rule these out.

**Necrotizing enterocolitis (NEC**): Typically affecting premature neonates, NEC presents with systemic instability, bloody stools, and radiological evidence of bowel wall gas. None of these were present in our patient.

**Congenital anomalies of the kidney and urinary tract (CAKUT**): These structural anomalies predispose to infections and pyonephrosis. Although not diagnosed antenatally, follow-up imaging post-discharge was advised to identify any underlying abnormalities, such as posterior urethral valves or vesicoureteral reflux. Table 1 summarizes these differential diagnoses.

**Table 1:** Differential diagnosis of neonatal pyonephrosis.

Differential diagnosis	Typical age group	Symptoms	Imaging findings	Key differentiating features
Hydronephrosis (Non-infective)	All ages, congenital or acquired	Usually asymptomatic; abdominal mass or UTI if severe	Dilated renal pelvis/calyces without internal echoes	No signs of infection; no debris or pus; normal labs
Renal or perinephric Abscess	Infants to adolescents	Fever, flank pain, palpable mass, irritability	Hypoechoic or complex mass adjacent to the kidney	Localized abscess with systemic symptoms; distinct on USG
Neonatal sepsis of unknown source	Neonates and infants	Fever, poor feeding, lethargy, respiratory distress	Normal or non-specific findings	Systemic signs without localized urinary tract findings
GI causes (e.g., Hirschsprung’s, malrotation)	Neonates and young infants	Vomiting, abdominal distension, failure to thrive	Air-fluid levels, dilated loops on abdominal X-ray/USG	GI origin signs like bilious vomiting or abnormal bowel sounds
NEC	Premature and low birth weight neonates	Abdominal distension, bloody stools, apnea, temperature instability	Pneumatosis intestinalis, portal venous gas on imaging	GI bleeding and radiographic bowel wall changes
CAKUT	Prenatal to infancy	Often asymptomatic early; UTI, poor stream, palpable mass later	Hydronephrosis, dilated ureters, abnormal bladder wall on USG	Confirmed postnatally by voiding studies or recurrent UTI

This structured comparison helped to rule out alternative possibilities and focus the diagnosis on pyonephrosis, which was confirmed through imaging and microbiological culture.

#### Microbiological insights

*Acinetobacter baumannii* is a gram-negative opportunistic pathogen, commonly associated with hospital-acquired infections and noted for its multidrug resistance. In our case, *A. baumannii* demonstrated resistance to piperacillin but remained sensitive to cefotaxime and amikacin. This highlights the importance of relying on local antimicrobial resistance patterns and drug sensitivity data when selecting empiric therapy, particularly in low-resource settings where multidrug-resistant organisms are increasingly encountered. Its isolation in a previously unexposed neonate suggests environmental or community origin, possibly related to poor hygiene or contaminated water. The sensitivity to cefotaxime and amikacin guided effective treatment, underscoring the importance of culture-directed therapy. The rising prevalence of *Acinetobacter* species in neonatal intensive care unit settings mandates vigilance and local antibiogram surveillance.

#### Treatment and outcome

Early drainage of pus and decompression of the collecting system is the cornerstone of pyonephrosis management. In our case, emergency percutaneous nephrostomy enabled rapid resolution of infection, avoidance of nephrectomy, and preservation of renal function. Antibiotic choice was tailored after culture sensitivity. Notably, despite the presence of a multidrug-resistant organism, the child responded favorably due to timely intervention.

Traditional management often involved nephrectomy in cases of non-functioning kidneys or delayed presentation. However, nephron-sparing strategies like image-guided nephrostomy are now preferred, especially in neonates, where renal preservation is crucial for long-term growth and development.

#### Challenges in low-resource settings

The case highlights the unique obstacles faced in resource-limited hospitals: lack of access to pediatric CT or MRU, limited microbiological infrastructure, and narrow antibiotic options. Despite these limitations, timely diagnosis using US, percutaneous drainage, and rational use of available antibiotics ensured a positive outcome. Such success stories reinforce the need for training general physicians and pediatricians in essential point-of-care imaging and emergency procedures.

#### Comparative literature review

Few reports in the literature describe neonatal pyonephrosis, underscoring its rarity. In a case reported by Patel *et al*, a term neonate presented with methicillin-resistant staphylococcus aureus-induced pyonephrosis, managed successfully with percutaneous nephrostomy and targeted antibiotics^[^1^]^. Similarly, Cheng *et al* analyzed multiple pediatric cases of renal abscesses and severe urinary infections, noting varied pathogens including *Staphylococcus aureus, Escherichia coli*, and *Klebsiella pneumoniae*, with mixed outcomes depending on the timeliness of drainage and choice of antibiotics^[^3^]^.

Compared to these, our case is distinctive in several respects:

1. the young age of the patient (48 days old),
2. identification of *A. baumannii* as the causative organism – a multidrug-resistant pathogen more often seen in hospital-acquired infections, and
3. favorable recovery without the need for nephrectomy, using only ultrasound guidance and basic culture facilities available in a low-resource setting.

This case reinforces that even under technological constraints, adherence to early suspicion, drainage, and culture-based therapy can lead to successful outcomes in neonatal urological emergencies.

#### Recent advances

Recent advances in artificial intelligence (AI) have demonstrated promising applications in medical diagnostics and drug discovery. For example, the AlphaFold model has significantly advanced molecular biology research and drug development through machine learning–based protein structure prediction. Similarly, AI models using clinical imaging data have shown potential for improving personalized diagnosis and treatment strategies^[^10,11^]^. In the context of resource-limited settings, AI-assisted ultrasound image analysis may help automatically identify characteristic echoes of renal pelvic pus and estimate pus volume, potentially improving early diagnosis of pyonephrosis. Lightweight AI tools, including mobile-based diagnostic applications, could support clinicians in areas with limited radiological expertise. Furthermore, machine learning models based on local microbial databases may help predict antimicrobial resistance patterns, enabling faster and more accurate empirical antibiotic selection. Integration of telemedicine platforms with AI-based diagnostic tools may also facilitate multidisciplinary collaboration among pediatricians, radiologists, and microbiologists, improving care delivery in resource-constrained environments.

## Conclusion

This case demonstrates the successful use of ultrasound-guided percutaneous nephrostomy combined with targeted antibiotic therapy in the management of neonatal pyonephrosis in the absence of advanced imaging modalities such as CT. The case highlights the importance of rapid clinical suspicion, timely drainage, and antimicrobial therapy guided by microbial resistance surveillance. These strategies can help preserve renal function and achieve favorable outcomes even in low-resource healthcare settings.

## Patient perspective

When our baby became sick with fever, vomiting, and constant crying, we were terrified and did not understand what was happening. At just over a month old, he had never been ill before. We live in a village and initially thought it might just be gas or some common infection. When the swelling in his belly worsened, we brought him to the hospital. The doctors explained that he had a serious kidney infection and needed immediate treatment. We were shocked when they said they would drain pus from his kidney, but they kept us informed every step of the way. Within a few days, we saw him improve; his fever went down, he started feeding again, and he looked like our baby once more. We are grateful for the care he received, especially since we could not afford expensive tests. The doctors acted fast, used simple tools like an ultrasound, and treated him with the right medicines. We thank the entire team for saving his kidney and his life.

## Data Availability

No data sets were used in the production of this report.
